# Modeling the Non-Stationary Climate Dependent Temporal Dynamics of *Aedes aegypti*

**DOI:** 10.1371/journal.pone.0064773

**Published:** 2013-08-20

**Authors:** Taynãna C. Simões, Cláudia T. Codeço, Aline A. Nobre, Álvaro E. Eiras

**Affiliations:** 1 Department of Epidemiology and Quantitative Methods in Health - DEMQS-ENSP/Fiocruz, Rio de Janeiro, RJ, Brazil; 2 Scientific Computing Program - PROCC/Fiocruz, Rio de Janeiro, RJ, Brazil; 3 Scientific Computation Program - PROCC/Fiocruz, Rio de Janeiro, RJ, Brazil; 4 Department of Parasitology - Institute of Biological Sciences, Federal University of Minas Gerais, Belo Horizonte, MG, Brazil; SUNY College of Environmental Science and Forestry, United States of America

## Abstract

**Background:**

Temperature and humidity strongly affect the physiology, longevity, fecundity and dispersal behavior of *Aedes aegypti*, vector of dengue fever. Contrastingly, the statistical associations measured between time series of mosquito abundance and meteorological variables are often weak and contradictory. Here, we investigated the significance of these relationships at different time scales.

**Methods and Findings:**

A time series of the adult mosquito abundance from a medium-sized city in Brazil, lasting 109 weeks was analyzed. Meteorological variables included temperature, precipitation, wind velocity and humidity. As analytical tools, generalized linear models (GLM) with time lags and interaction terms were used to identify average effects while the wavelet analysis was complementarily used to identify transient associations. The fitted GLM showed that mosquito abundance is significantly affected by the interaction between lagged temperature and humidity, and also by the mosquito abundance a week earlier. Extreme meteorological variables were the best predictors, and the mosquito population tended to increase at values above 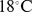 and 54% humidity. The wavelet analysis identified non-stationary local effects of these meteorological variables on abundance throughout the study period, with peaks in the spring-summer period. The wavelet detected weak but significant effects for precipitation and wind velocity.

**Conclusion:**

Our results support the presence of transient relationships between meteorological variables and mosquito abundance. Such transient association may be explained by the ability of *Ae. aegypti* to buffer part of its response to climate, for example, by choosing sites with proper microclimate. We also observed enough coupling between the abundance and meteorological variables to develop a model with good predictive power. Extreme values of meteorological variables with time lags, interaction terms and previous mosquito abundance are strong predictors and should be considered when understanding the climate effect on mosquito abundance and population growth.

## Introduction

The mosquito *Aedes aegypti* is the main vector of dengue fever, an important arbovirosis present in most tropical countries. The effect of climate on the population dynamics of *Ae. aegypti* has been the subject of several studies [1]–[8]. The dependence of mosquito development, survival and behavior on air temperature, air humidity, rainfall and wind speed is well established [1]. In general, rainfall is expected to positively affect the mosquito abundance through the creation of new breeding sites. Heavy precipitation, on the other hand, may have an opposite flushing effect. Temperature affects the mosquito's development as well as its survival and fecundity. Wind may drive passive dispersal and/or induce the suppression of the flying activities, affecting feeding and egg-laying activities. In turn, the air relative humidity directly influences survival, feeding habits and dispersal [1], [9], [10].

Contrasting with the strong responses of *Ae. aegypti* to meteorological variables in experimental studies, the statistical associations measured between time series of mosquito abundance and meteorological variables can be weak and contradictory, showing sometimes positive or negative effects. Most studies associating *Ae. aegypti* abundance and climate have relied on larval indices that are collected every one to three months, using regression analysis as the main analytical tool [8], [11]. Overall, these studies have identified positive associations with temperature, precipitation and relative humidity at several lags as expected from the theory. However, their results are hard to compare as the analytical methodology used is highly variable. For example, it is not explicit if interaction terms were evaluated or how the selection of variables was carried out.

More recently, more detailed studies became possible due to the introduction of traps into adult mosquito surveillance systems. With traps, entomological indices can be collected more frequently, generally every week. The resulting indices are more informative for dengue surveillance as they measure directly the population involved in the transmission cycle: the adult female population [12]. Another advantage of trap indices is their resolution at a one-week time scale, which is important for studying the effect of climate on a population whose generation time is approximately 3–4 weeks long [1]. Using BG-Sentinel in Cairns, Australia, a trap that attracts females seeking humans for blood feeding, Azil et al. (2010) [2] analyzed a 134 week time series of female mosquito captures using regression. They found positive effects of minimum and average temperature (lag 0 weeks) and relative humidity (lag 2 weeks). Honório et al. (2009) [4] analyzed an 80 week long series of sticky trap collections, in Rio de Janeiro, Brazil, finding a nonlinear association with temperature and a weak association with precipitation. These studies highlight the relevance of short term meteorological events, in the order of weeks.

In the present study, we contribute to this problem by analyzing a 109 week long time series of sticky trap data from an *Ae. aegypti* surveillance system implemented in the municipality of Governador Valadares (MG, Brazil) where the climate is sub-humid tropical. In contrast to previous studies, we propose the combination of two time series techniques for further understanding the short and long term associations between climate and mosquito abundance. First, a linear model was fitted to the data, producing a climate-based model with good forecasting capacity. Secondly, a multi-resolution wavelet method was applied to identify transient patterns of association between the entomological indices and meteorological variables. With this combined analysis, we were able to characterize the global and local (transient) effects of meteorological variables on mosquito abundance and discuss possible implications for the development of climate based forecast models.

## Methods

### Ethics Statement

All traps used in this study were installed by the official staff of the city dengue control program during the routine determination of the house infestation index. In these activities, no written consent is required, or any formal permission for mosquito collection. Therefore, consent is oral and informal. Furthermore, during these routine activities of surveillance, field workers do not record any personal information from householders. Data presented in this manuscript cannot be used to identify specific houses, so the anonymity of householders is guaranteed.

### Study area

**Figure 1:**
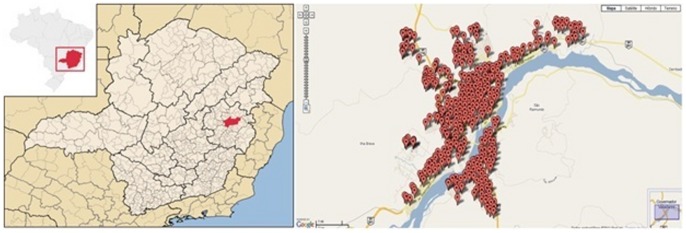
Study Area. Map of the municipality of Governador Valadares and the locations of set traps.

Using traps to capture ovipositing mosquitoes, a longitudinal study was conducted as part of a vector surveillance program in the urban area of the Governador Valadares municipality (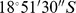, 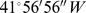), a medium-sized city located in the Eastern region of Minas Gerais state, Brazil (Figure 1) [12]. Governador Valadares has an estimated population of 263,274 inhabitants and a population density of 112.1 inhabitants/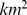. The climate is sub-humid tropical with a rainy season from October to April and a dry season from May to September. The average annual temperature varies around 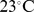, with a minimum of 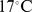 and a maximum of 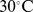. The average relative humidity is 70%, with predominant winds in the northeast direction [13].

### Data sources and management

A total of 425 MosquiTRAP were set throughout the study area (Figure 1), placed 200–250 meters apart and monitored weekly during 90 weeks (epidemiological weeks 11/2009 to 48/2010). MosquiTRAP*™* (Ecovec S.A., Belo Horizonte, Brazil) [12] is a sticky trap that captures gravid *Ae. aegypti* seeking places to oviposit [12]. For operational reasons, a few traps were not checked every week and the data collection process was not completely homogeneous. Considering the variability in the number of observations per week, capture data was summarized as mean capture rate per trap per week calculated as the ratio between the total number of mosquitoes captured during the week and the number of positive traps. Moreover, an additional 19-weeks time series was used to assess the predictive ability of the fitted linear generalized model. The trap data was kindly provided by the biotechnology “spin-off” company Ecovec S.A.. The data will be freely available if requested to authors.

Meteorological data in the form of average weekly accumulated rainfall (mm); minimum, average and maximum temperature (°C); minimum, average and maximum relative air humidity (%); and wind velocity (m/s) were obtained at Ministry of Health Environmental Information System - SISAM website (http://sisam.cptec.inpe.br/msaude/).

### Generalized Linear Models (GLM)

The average effects of the meteorological variables on weekly mosquito abundance were estimated using generalized linear models with Negative Binomial distribution using the logarithmic link function [14]. Since the variance of the mosquito abundance distribution (6393) was much greater than its mean (210), characterizing over-dispersion, the Negative Binomial probability distribution was chosen instead of the Poisson distribution, the typical probability distribution for modeling counting data. The response variable was the total number of female *Ae. aegypti* mosquitoes on the week 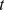 (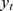), modeled as:
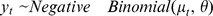


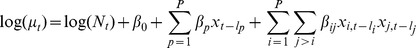
where *μ_t_* is the average of the response variable on the week 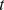, 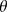 represents the extra variability parameter of the data, and the 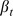
's are the parameters that measure the effects of the 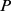 lagged (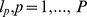) meteorological variables and the autoregressive term (mosquito abundance per trap on the previous week). The parameters 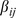
's represent the effects of the meteorological variables interaction terms. The term 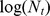 corresponds to the model offset, representing the Natural logarithm of the number of traps observed in week 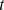. With the introduction of the offset term, one corrects for the nonhomogeneity of the capturing process.

The modeling strategy was as following: First, the effect of each meteorological variable, lagged up to 4 weeks (lags 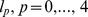), on mosquito abundance was assessed individually at a level of 5% significance. This range of lags was chosen based on the lifespan of the adult mosquito that can vary from 2 to 4 weeks, depending upon environmental and climatic conditions. Second, multivariate analysis was carried out by the introduction of one by one meteorological variables retained in the previous step, ranked in descending order of significance with the response variable (two more significative lagged variables). More than two variables had not been included into models to avoid multicollinearity. At last, the autoregressive term (mosquito abundance per trap in the previous week) was included in the model. Models were tested with possible interaction terms between the lagged meteorological covariates included. In all models, the intercept was removed from the linear predictor. We presented the best fitted models for all combinations of interactions between temperature and humidity (nine combinations - each temperature variable with each humidity variable), chosen by the lowest Akaike Information Criterion - AIC e Deviance impact [15]–[17].

Residual analysis was carried out, including an assessment of the presence of temporal structure in the residuals using autocorrelation (ACF) and partial autocorrelation functions (PACF) [14], [15]. The model predictability for forecasts of “out-of-fit” data was evaluated by the projection of the predicted values (and the 95% confidence interval) for 19 weeks ahead of the fitted series. We calculated the Spearman's Rank Correlation coefficient (SRC) for assessing linear association between the forecasted and real time series [18].

### Wavelet Analysis

The wavelet transform is a linear operation used in the analysis of signals, for the purpose of extracting relevant information from frequency variations, in addition to detecting important local temporal structures, as abrupt peaks and gaps. This technique also enables analysis of relations between two non-stationary series and location of intermittent oscillations, identifying gradual changes in the strength of exogenous variables [19]. The correlation may be analyzed through cross wavelet transform and wavelet coherence [20], [21]. The former exposes the regions of high variability in common for the two series, while the latter identifies regions where the two series oscillate on the same frequency. The most used transformation function is the Morlet wavelet, whose results can be interpreted as periods (wavelength) of the oscillatory components. Reviews of the method can be seen in Cazelles et al. [20] and Torrence and Compo [22].

The time series variance distribution may be represented by a bi-dimensional image, known as the wavelet power spectrum, which shows the amplitude of oscillatory components versus frequency scale, and how this amplitude varies over the time [20]. Monte Carlo methods and chi-squared tests are used to assess statistical significance of the observed variability, highlighting contour levels of 95% confidence in relation to standard power spectrum, the red noise or first order autoregressive process (Null Hypothesis) [20], [22]. The cone of influence (COI), represented by a parabola superimposed on the power spectrum, delimits the region free from edge effects (hatched areas in darker tone in the center of the spectrum) [20], [22]. Relative phase between variables are indicated by the angle of small arrows. Arrows pointing right indicate in-phase processes and arrows pointing left indicate out-phase processes [21], [22].

In this study, the wavelet analysis was used only as a complementary analysis to the GLM models. The visual analysis of the wavelet spectra allows checking in which time intervals, the associations are significant. The wavelet spectra of the meteorological variables and the interaction terms are presented. The interaction term was composed as the time series resulting from the multiplication between the time series of meteorological variables interacting.

Prior to the wavelet analysis, the natural logarithmic transformation was applied to the mosquito abundance time series as well as to the meteorological variables in order to obtain approximately normal distributions (improving chi-squared test's accuracy). The series were standardized (zero mean and standard deviation one) for better comparability. Note that scale changes of the variables do not change the appearance of the wavelet spectrum. All analyses were conducted using the R environment [23] and Matlab, with the routines available at the sites http://paos.colorado.edu/research/wavelets/ by Torrence and Compo [22] and http://www.pol.ac.uk/home/research/waveletcoherence/ by Grinsted et al. [21].

## Results

**Figure 2:**
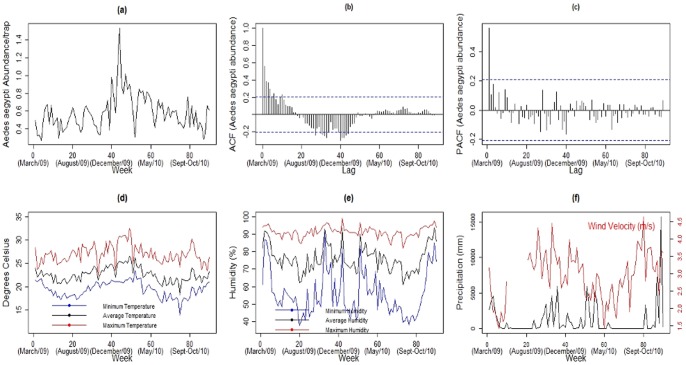
Descriptive plots. Times series (a), autocorrelation function - ACF (b) e partial autocorrelation function - PACF (c) of *Ae. aegypti* abundance per trap; minimum, average and maximum temperature (d), minimum, average and maximum relative air humidity (e), precipitation (mm) and wind velocity (m/s) (f).

A total of 18,959 *Ae. aegypti* female mosquitoes were captured during the study period, with an average of 210.7 per week, ranging from 61 to 581 mosquitoes per week. This corresponded to an average mosquito abundance per trap of 0.58 (minimum = 0.27, maximum = 1.53 mosquitoes per week per trap). The time series of mosquito abundance per trap displays non-stationary features (Figure 2a) with an increasing trend of infestation up to week 44, in the summer, followed by a slight downward trend. An outlier of 1.53 mosquitoes per trap was observed on the week 45. The autocorrelation (ACF), and partial autocorrelation functions (PACF) (Figures 2b and 2c) show the presence of temporal structure. The ACF shows that some lags exceed the limits built on the hypothesis that the data are independent, and the PACF presents a slight seasonal pattern.

The meteorological conditions during the study were typical for the region. The temperature averaged 19.36°C (minimum temperature), 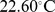 (average temperature), and 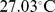 (maximum temperature), varying from 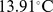 to 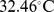. Thus, it did not show great variability over the period analyzed (Figure 2d). The relative air humidity was higher during the warmer months and averaged 55.93% (minimum humidity), 76.22% (average humidity), and 91.29% (maximum humidity) (Figure 2e), with the minimum humidity presenting the greatest variability. The records of temperature and humidity were consistent with previous years, with no anomalous events. The rainfall averaged 61 mm, with a peak of 15760.2 mm in the end of the spring of 2010 (November). There was no rain in 35 of the 90 weeks, with the dry period corresponding to the winter months (May–August) (Figure 2f). The average wind speed was low, 2.9 m/s, with lower values during fall in early May (Figure 2f).

### Generalized Linear Models (GLM)

The temperature and humidity lags most associated with mosquito abundance can be seen in Table 1, listed in descending order of statistical association with the response variable. The first order autoregressive term - AR(1) was also significant. The precipitation and wind were not significant, at any lag (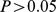).

**Table 1:** Univariate models.

Variable (lag)	Estimate	Standard Error	p-value
AR(1)	0.8727	0.1427	<0.0001
Maximum Temperature (0)	0.0799	0.0149	<0.0001
Average Temperature (0)	0.0888	0.0192	<0.0001
Minimum Temperature (4)	0.0697	0.0181	0.0001
Minimum Humidity (0)	−0.0070	0.0026	0.0061
Maximum Humidity (2)	0.0287	0.0110	0.0094
Average Humidity (2)	0.0107	0.0046	0.0187

Estimates of individual effects of lagged variables (lag) on mosquito abundance/week/trap according to the generalized linear model.

The best model was chosen from nine combinations of models with temperature and humidity interactions at different lags, using the Akaike Information Criterion - AIC (Table S1). The air humidity at 2 weeks lag was the most significant term in all models, followed by the 0-lag maximum temperature, and the 4 weeks lag average and minimum temperatures.

The best model was the one containing the AR(1) term, the 4-lag minimum temperature, the 2-lag minimum humidity and the interaction between the last two. The Figure S1 and the Table S3 show a gradual improvement in the fit from the model with only the autoregressive term (Figure S1a - AIC = 1053.46, SCR = −0.0107), the model with only meteorological variables (Figure S1b - AIC = 947.68, SCR = 0.5202) and the best model (Figure S1c - AIC = 922.27, SCR = 0.6610). Figure S2 shows the goodness-of-fit and residuals analysis plots. From these plots, one can observe a strong correlation between the observed and fitted time series, and that the fitted model complies with the GLM assumptions of normality, linearity and homoscedasticity, as well as the lack of autocorrelation structure in the residuals. Table 2 shows the estimated effects, variability and statistical significance of each term in the final model. The significant interaction term between minimum temperature (lag 4) and minimum humidity (lag 2) implies that the effect of one meteorological variable on the abundance changes, according to the values that the other variable takes. Figure S3 shows how the interaction between minimum air humidity and minimum temperature affects mosquito abundance, indicating that effects change direction at values below or above 43.6% for minimum humidity and 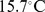 for minimum temperature. Table S2 further shows this threshold as estimated by the other nine models.

**Table 2:** Best fitted model.

Model : AR(1)+Minimum Temperature+Minimum Humidity+Interaction Term (Minimum Temperature*Minimum Humidity)			
Variable	Estimate	Standard Error	p-value
Mosquito abundance (*lag* = 1)	0.8344	0.1481	1.78e-08
Minimum Temperature (*lag* = 4)	−0.0714	0.0103	4.05e-12
Minimum Humidity (*lag* = 2)	−0.0257	0.0066	0.0001
Interaction Term	0.0016	0.0003	8.06e-09
Overdispersion Parameter	19.1100	3.1600	

Estimates of fitted model with climatic variables and interaction term effects on mosquito abundance/week/trap.

**Figure 3:**
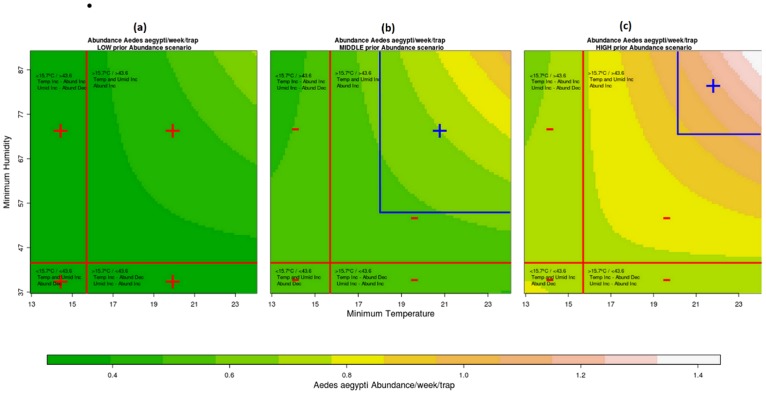
Interaction Heat Maps. Heat maps graphs of the *Ae. aegypti* abundance/week/trap growth with change in values of Minimum Temperature and Minimum Humidity, for LOW (a), MIDDLE (b) and HIGH (c) mosquito abundance in the preceding week. The quadrants formed by the red lines limited by the thresholds (15.7°C and 43.6%) separate the shift effects on mosquito abundance, that can increase (Inc) or decrease (Dec) as the temperature and humidity values increase. Plus sign indicates population growth and minus sign indicates no population growth. The ascending color scale represents the effect on the abundance as the temperature and humidity increase. Blue lines delimit regions where population growth in MIDDLE and HIGH scenarios.

The best model was used to predict the expected environmental conditions associated with positive mosquito population growth. We compared three scenarios (Figure 3), by varying the mosquito density in the previous week (the AR(1) term). In the first scenario (LOW), when mosquito density in the previous week was low (0.111 mosquitoes/week/trap), the model predicted positive population growth independently of the meteorological conditions. In the second scenario (MIDDLE), with median mosquito density (0.56 mosquitoes/week/trap), population growth is expected only if minimum temperature is above 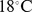 and minimum air humidity is above 55%. If mosquito abundance is high (scenario HIGH, with 0.99 mosquitoes/week/trap), on the other hand, conditions for positive population growth are restricted to minimum temperature above 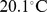 and minimum air humidity above 72%. Figure S4 shows the model capacity to predict the mosquito abundance in the (non-fitted) following 19 weeks. We can observe the good performance of the fitted model for forecasts of “out-of-fit” data (Spearman's Rank Correlation coefficient (SRC) = 0.72).

### Wavelet Analysis

**Figure 4:**
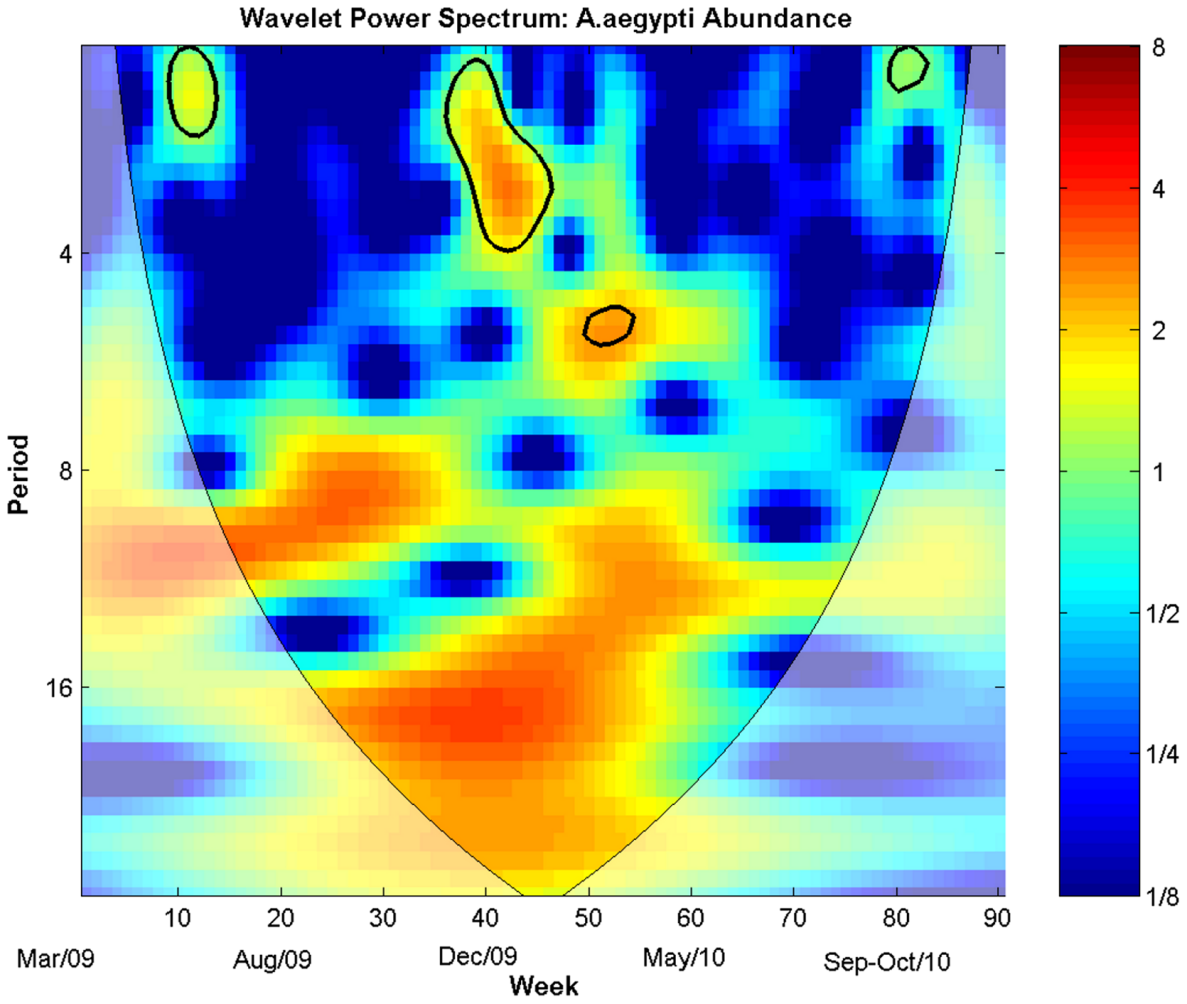
Mosquito Abundance Power Wavelet Spectrum. Power wavelet spectrum of *Ae. aegypti* abundance/week/trap. Times series was log-transformed and normalized. Colors code for increasing spectrum intensity, from blue to red. Black bolder contours show statistically significant area (threshold of 95% confidence interval). The black curve delimits the cone of influence (region not influenced by edge effects). Period scale is in weeks. The y-axis is on a base 2 logarithmic scale.

The power spectrum of the mosquito time series (Figure 4) presents sparse areas with significant oscillations. The most dominant peak occurs with a period of 1–4 weeks at the end of spring and onset of the 2009 summer (weeks 37 to 47) coinciding with the largest abundance peak in the time series (see Figure 2a). The spectrum shows a weaker but still significant 2-week peak in the fall (10–15 weeks).

**Figure 5:**
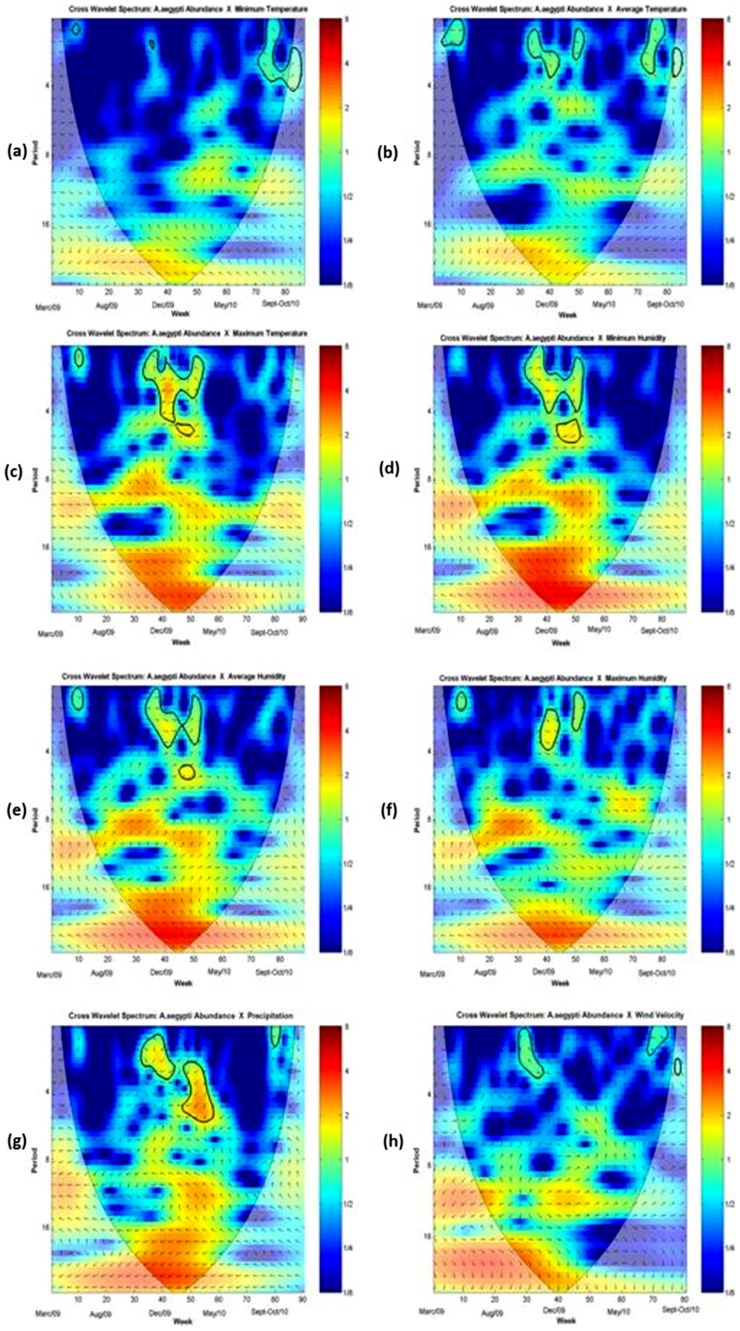
Meteorological variables and mosquito abundance Cross Wavelet Spectrums. Cross wavelet spectrum of *Ae. aegypti* abundance/week/trap *versus*: Minimum Temperature (a); Maximum Temperature (c); Average Humidity (e); Precipitation (g). Average Temperature (b); Minimum Humidity (d); Maximum Humidity (f); Wind Velocity (h). Times series were log-transformed and normalized. Colors code for increasing spectrum intensity, from blue to red. Black bolder contours show statistically significant area (threshold of 95% confidence interval). The black curve delimits the cone of influence (region not influenced by edge effects). Period scale is in weeks. The y-axis is on a base 2 logarithmic scale. The black arrows represent the relative phase relationship (anti-clockwise direction starting at the west-east direction). In all graphs, the first series is the mosquito abundance and the second series is a meteorological variable: 0°: both series are in-phase; 45°: the second series is 1/8 of period ahead of the former, 90°: 1/4 of the period ahead; 135°: 3/8 of the period ahead; 180°: the series are out-phase; 225°: the second series is 3/8 of the period behind; 270°: 1/4 of the period behind, 315°: 1/8 of the period behind.

**Figure 6:**
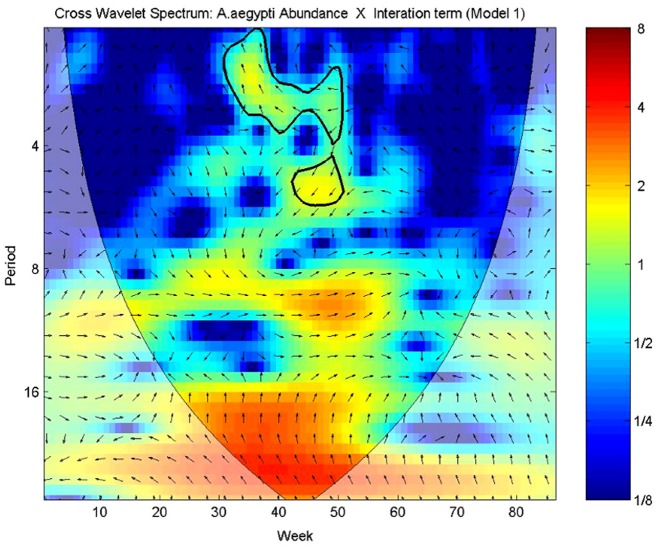
Interaction terms and mosquito abundance Cross Wavelet Spectrums. Cross wavelet spectrum of *Ae. aegypti* Abundance/week/trap *versus*: First column: Minimum Temperature; Maximum Temperature; Average Humidity; Precipitation. Second column: Average Temperature; Minimum Humidity; Maximum Humidity; Wind Velocity. Times series were log-transformed and normalized. Colors code for increasing spectrum intensity, from blue to red. Black bolder contours show statistically significant area (threshold of 95% confidence interval). The black curve delimits the cone of influence (region not influenced by edge effects). Period scale is in weeks. The y-axis is on a base 2 logarithmic scale. The black arrows represent the relative phase relationship (anti-clockwise direction starting at the west-east direction). In all graphs, the first series is the mosquito abundance and the second series is a meteorological variable: 0°: both series are in-phase; 45°: the second series is 1/8 of period ahead of the former, 90°: 1/4 of the period ahead; 135°: 3/8 of the period ahead; 180°: the series are out-phase; 225°: the second series is 3/8 of the period behind; 270°: 1/4 of the period behind, 315°: 1/8 of the period behind.

Figures 5 and 6 show the cross wavelet spectra between mosquito abundance and the meteorological variables. Overall, the associations were intermittent during the study period (Figure 5). The effects of minimum and average temperature are more stable, although the effect of the former is very weak. Minimum temperature presented greater association with mosquito abundance during the winter-spring of 2010, when both time series were at their lowest (Figure S6a). Mosquito abundance presented more association with minimum humidity during the spring-summer seasons of 2010 (Figure S6b), the same period when the joint association of minimum temperature and minimum humidity with the mosquito abundance was more significant (Figure S6c). The strongest crude effect was observed between mosquito abundance and maximum temperature. Contrasting with the regression model, the wavelet detected significant transient associations between precipitation and wind velocity.

The wavelet coherence spectra (Figure S5) show that there are common regions of variability between mosquito abundance and the meteorological variables some with high power (0.6 to 1.0), while others with low energy (which are not present in the cross wavelet spectrum).

## Discussion

In this study, we demonstrate that a generalized linear model at the week time scale with minimum temperature and minimum humidity as covariates is a good predictive model for the abundance of *Ae. aegypti* in a sub-humid tropical city characterized by well defined dry and wet seasons. The model did not only presented a good fit to the historical data but was also able to forecast 19 weeks of data with good precision. This result is valuable for vector surveillance in dengue endemic areas with similar climate. We also observed that the quality of the model was highly depended on the inclusion of an AR(1) term and the interaction between the two meteorological variables. The best fitted model, with AR(1), minimum air temperature (lag 4 weeks), minimum humidity (lag 2 weeks) and the interaction between these two meteorological variables, corroborates with Azil et al. [2], who observed significant main effects of the average temperature and average humidity at two week lag, in Cairns, Australia. However, their model did not contain the interaction term.

Despite the natural perception that there is interaction between temperature and humidity, climate models generally do not explicit test such terms. None of the models we surveyed in the literature presented interaction terms. Costa et al. [6] evaluated the impact of small variations in temperature and humidity on the reproductive activity and survival of *Ae. aegypti*. It was shown that, independent from humidity, an increase in temperature led to a decrease in the number of eggs and a decrease in the egg-laying time. Low temperature and high humidity resulted in greater survival of mosquitoes and greater egg productivity, compared to a scenario of high temperature and low humidity. When humidity was low, there was a reduction in egg fertility as temperature increased. For temperatures greater than 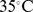, it was clearly shown that the decrease in mosquito population was influenced jointly by temperature and humidity.

The interaction between temperature and humidity produced a more informative model for mosquito surveillance. The model predicted population growth at temperature and humidity values above 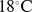 and 54% in a scenario where in the previous week, mosquito abundance was average, and above 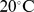 and 72% in a scenario where mosquito abundance was already high. These thresholds can provide empirical information regarding the environmental conditions that are favorable for *Ae. aegypti* proliferation in this setting. Experimental observations where mosquitoes develop under controlled conditions show that below such temperatures, development is delayed and survival is greatly reduced [24]. Low humidity also has an important deleterious effect on survival [6] fecundity and fertility [25].

Despite the good fit of the linear model, further investigation using wavelet techniques showed that the association between climate and mosquito abundance is not constant, as assumed by the linear model. For example, a biological explanation for intermittent associations is the observation that mosquitoes are capable of finding refuge from harsh environmental conditions by hiding inside houses or avoiding activity during rainfall. Such behaviors can buffer part most of the climate dependent dynamics and produce only intermittent associations with meteorological variables when they assume values that are too extreme or persistent to be avoided [26].

Intermittence in the association between variables has consequences for the application of regression models. A time series is said to be non-stationary when its statistical properties, such as mean and variance, vary over time. When a temporal series contains dominant periodic components that vary in amplitude and frequency, and this heterogeneity results in high variability on different frequency scales, rendering the series non-stationary, classical statistical analysis techniques may be inadequate. This characteristic is especially evident in epidemiological time series with strong autocorrelation and variation [20], [22]. Conventional statistical methods may fail to test the relation between variables when discontinuous or non-stationary associations are present [27], [28].

Wavelet technique enables the identification of transient behaviors in time series, separating oscillations with periods of shorter duration from those with longer duration. It has been used in several fields such as meteorology, geophysics, statistics and signal processing. In the context of dengue fever, several studies have been done, all of them agreeing on its complex association with climate. For instance, Cumming et al. [29] identified travelling waves of dengue epidemics in Bangkok from 1983 to 1997 using Empirical Mode Decomposition, a method analogous to wavelet analysis. Cazelles et al. [27] found high-yet-discontinuous and transient association between El Niño, precipitation and dengue epidemics whereas Nagao and Koelle [30] used wavelet to identify a shift in the frequency and the dominant periodic components of the largest dengue epidemic in Thailand. Interesting, Johansson et al. [31] analyzed the relation between El Niño, local weather, and dengue incidence in Puerto Rico, Mexico and Thailand showing that ENSO was transiently associated with temperature and dengue incidence on multi-year scales. Thai et al. [32] detected seasonal and inter-annual cycles in the incidence of dengue, varying over the time and space and relations with ENSO. Vazquez-Prokopec et al. [19] applied spatial wavelets and other techniques to describe spread of DENV-2 in the city of Cairns (Australia). Chaves and Kitron [21] used multi-scale analysis to show that relative air humidity is more significant at intermediary time scales, facilitating the movement of fertilized females and access to egg-laying sites. In another example, Chaves et al. [7] present results from simple mathematical models and cross wavelet to demonstrate that the density-dependent regulation was strong in *Ae. aegypti* population in Puerto Rico and Thailand, and that the population is more sensitive to climate variables with low kurtosis.

We believe that our qualitative results (non-stationary, transient dynamics) are robust to the limitations posed by the limited sample size. However, proper characterization of the seasonal and other periodic patterns likely to be present in the entomological time series will require a longer time series. This may result in less robust classifications of inter-weekly changes of variability analyzed using the wavelet technique. Another potential source of imprecision derives from the nature of the data collection process itself. As part of a nonscientific surveillance program, the collection process was vulnerable to irregular sampling effort. This irregularity was in part taken into account in the statistical analysis by the introduction of the offset term in the model. Future analysis should address longer time series. For these studies, we stress the importance of considering not only standard linear models, but also exploratory methods for transient patterns such as the wavelet. Another important constraint of this study is the exclusion of other determinants of mosquito dynamics, besides climate. It is well known that due to its highly domestic nature, *Ae. aegypti* dynamics is affected by urbanization, socioeconomic factors, sanitation conditions and vector control measures which should also be incorporated into predictive temporal models [1], [10], [31], [33].

One goal of entomological surveillance is to provide early warning for dengue outbreaks. In Brazil, as in many countries, surveillance is based on household larval surveys carried out three to four times a year. This approach is very time and labor intensive as well as prone to inaccuracy due to its dependence on human homogeneous sampling effort. These problems have led, to the emergence of alternative monitoring systems, based on georeferenced traps to sample egg and adult mosquitoes [5], [12], [23], [34]. Statistical methods that monitor possible abnormalities in the increase of these rates of infestation are important, distinguishing between scenarios within expectations, and a situation that calls for immediate control activities.

The findings of this study highlight the importance of jointly analyzing average effects and non-stationary associations of meteorological variables on mosquito abundance over the time, using terms of interaction at their extreme values. The results can be integrated into existing trap based surveillance systems for *Ae. aegypti* and be useful to the future development of warning systems based on climatic elements for prevention and control of dengue epidemics.

## Supporting Information

Figure S1 Goodness-of-fit for gradual fits of the best model. Graphs of overlapping of values observed (series in red) of mosquito abundance in the 90 weeks and fitted values (series in blue) of the model with only the AR(1) term (a), the model with only meteorological variables (b) and the best fitted model (The dashed lines correspond to confidence intervals of 95% of fitted values).
(TIF)

Figure S2 Goodness-of-fit and Residuals Analysis plots. First column: Graph of overlapping of values observed (series in red) of mosquito abundance in the 90 weeks and fitted values (series in blue) from the best fitted model (The dashed lines correspond to confidence intervals of 95% of fitted values); Residuals versus Fitted values plot; Residuals versus Leverage values plot. Second column: Mosquito abundance/trap observed versus fitted abundance/trap plot; Residuals Q-Q plot; Residuals ACF.
(TIF)

Figure S3 Interaction Plots. Graphs assessing effect of interaction between minimum temperature and minimum humidity on *Ae. aegypti* abundance/week/trap Fixed values of minimum temperature and minimum humidity corresponds to the quantile values of the distributions. The curves intersect at the humidity value of 43.6% (left) and 15.7°C (right).
(TIF)

Figure S4 Predictability model. Graph of overlapping of values observed (series in black) of number of mosquitoes in the 109 weeks and forecast values (series in blue) to “out-of-fit” data. The dashed lines correspond to confidence intervals of 95% of forecast values.
(TIFF)

Figure S5 Wavelet Coherence Spectrums. Wavelet Coherence spectrum of *Ae. aegypti* Abundance/week/trap *versus*: First line: Temperature (Minimum, Average and Maximum). Second line: Humidity (Minimum, Average and Maximum). Third line: Precipitation; Wind Velocity; Term Interaction (Model 1). Blue, low coherence; red, high coherence. The black bold contours show *α* = 5% significance level. The cone of influence (black curve) indicates the region not influenced by edge effects. Period scale is in weeks. The y-axis is on a base 2 logarithmic scale. The black arrows represent the relative phase relationship (anti-clockwise direction starting at the west-east direction). In all graphs, the first series is the mosquito abundance and the second series is a meteorological variable: 0°: both series are in-phase; 45°: the second series is 1/8 of period ahead of the former, 90°: 1/4 of the period ahead; 135°: 3/8 of the period ahead; 180°: the series are out-phase; 225°: the second series is 3/8 of the period behind; 270°: 1/4 of the period behind, 315°: 1/8 of the period behind.
(TIF)

Figure S6 Meteorological and Mosquito Abundance series plot. Times series plots of the minimum temperature (left), minimum humidity (center) and de interaction term (right) time series matched with the mosquito abundance/week/trap. Blue vertical lines delimit the time period in which the association was significant in the wavelet analysis.
(TIF)

Table S1 Model selection. Comparison of models with effects of the meteorological variables, autoregressive term and possible interactions of temperature and humidity (minimum, average and maximum) lagged weeks (lag) on mosquito abundance/week/trap.
(DOCX)

Table S2 Thresholds shift effects on mosquito abundance. Temperature and humidity thresholds shift effects on mosquito abundance/week/trap to the nine fitted models.
(DOCX)

Table S3 Process of fitting the best model. Estimates of the gradual fit of the best model.
(DOCX)
