# Supplementary figures and images for: Modeling the Non-Stationary Climate Dependent Temporal Dynamics of Aedes aegypti
Source: PLoS One. 2013 Aug 20;8(8):e64773. doi: 10.1371/journal.pone.0064773 (PMC3748059; doi:10.1371/journal.pone.0064773)

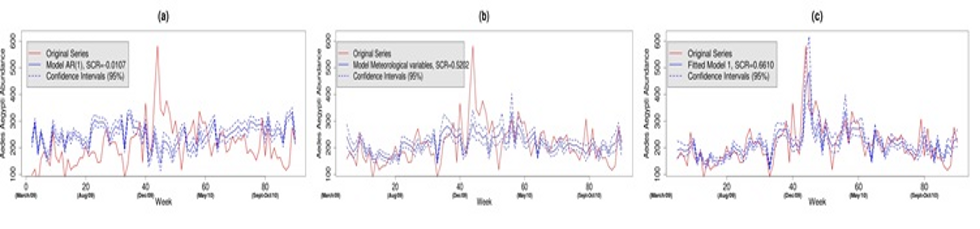

Supplement: Figure S1 — Goodness-of-fit for gradual fits of the best model. Graphs of overlapping of values observed (series in red) of mosquito abundance in the 90 weeks and fitted values (series in blue) of the model with only the AR(1) term (a), the model with only meteorological variables (b) and the best fitted model (The dashed lines correspond to confidence intervals of 95% of fitted values). (TIF) [file pone.0064773.s001.tif]

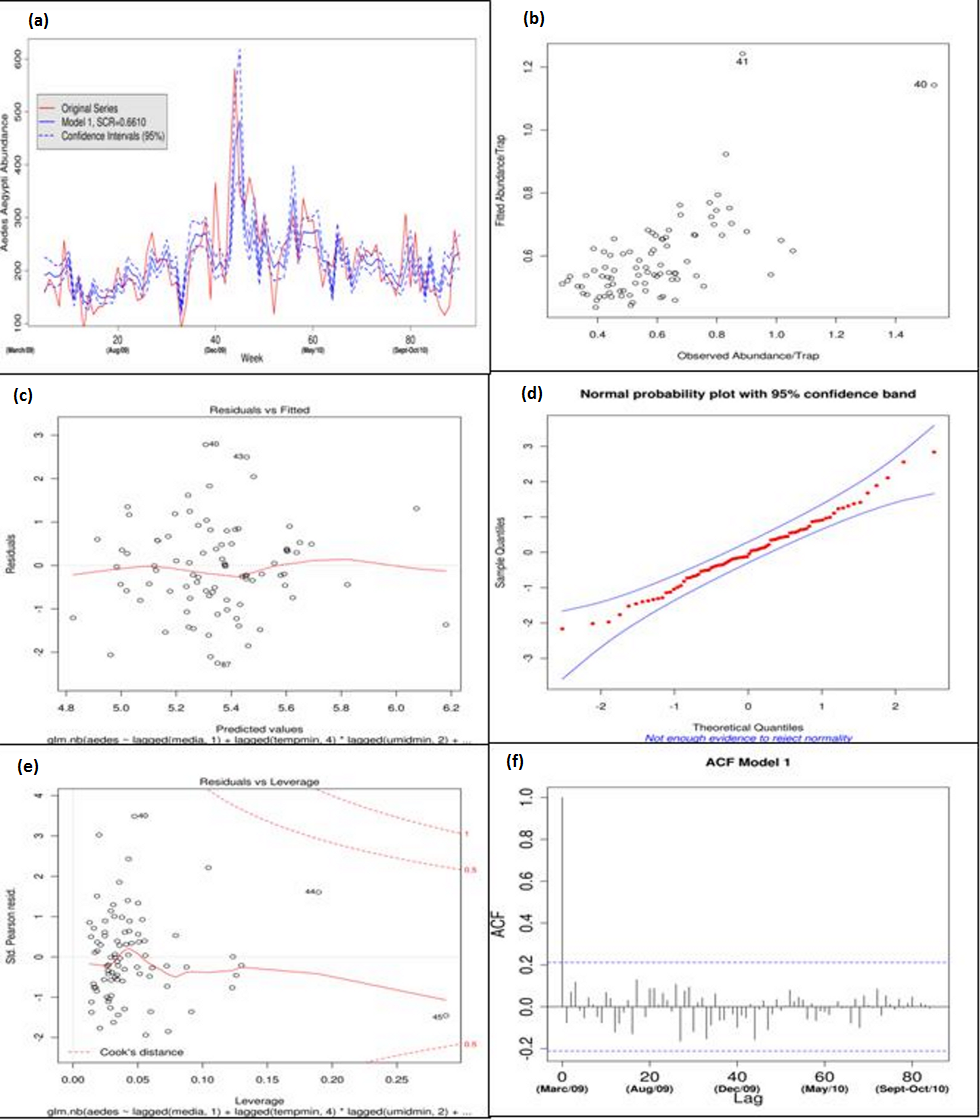

Supplement: Figure S2 — Goodness-of-fit and Residuals Analysis plots. First column: Graph of overlapping of values observed (series in red) of mosquito abundance in the 90 weeks and fitted values (series in blue) from the best fitted model (The dashed lines correspond to confidence intervals of 95% of fitted values); Residuals versus Fitted values plot; Residuals versus Leverage values plot. Second column: Mosquito abundance/trap observed versus fitted abundance/trap plot; Residuals Q-Q plot; Residuals ACF. (TIF) [file pone.0064773.s002.tif]

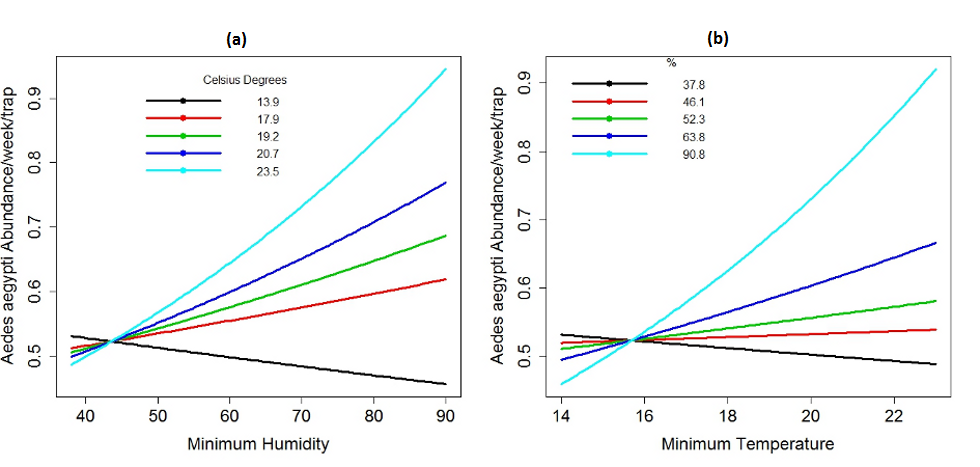

Supplement: Figure S3 — Interaction Plots. Graphs assessing effect of interaction between minimum temperature and minimum humidity on Ae. aegypti abundance/week/trap Fixed values of minimum temperature and minimum humidity corresponds to the quantile values of the distributions. The curves intersect at the humidity value of 43.6% (left) and 15.7°C (right). (TIF) [file pone.0064773.s003.tif]

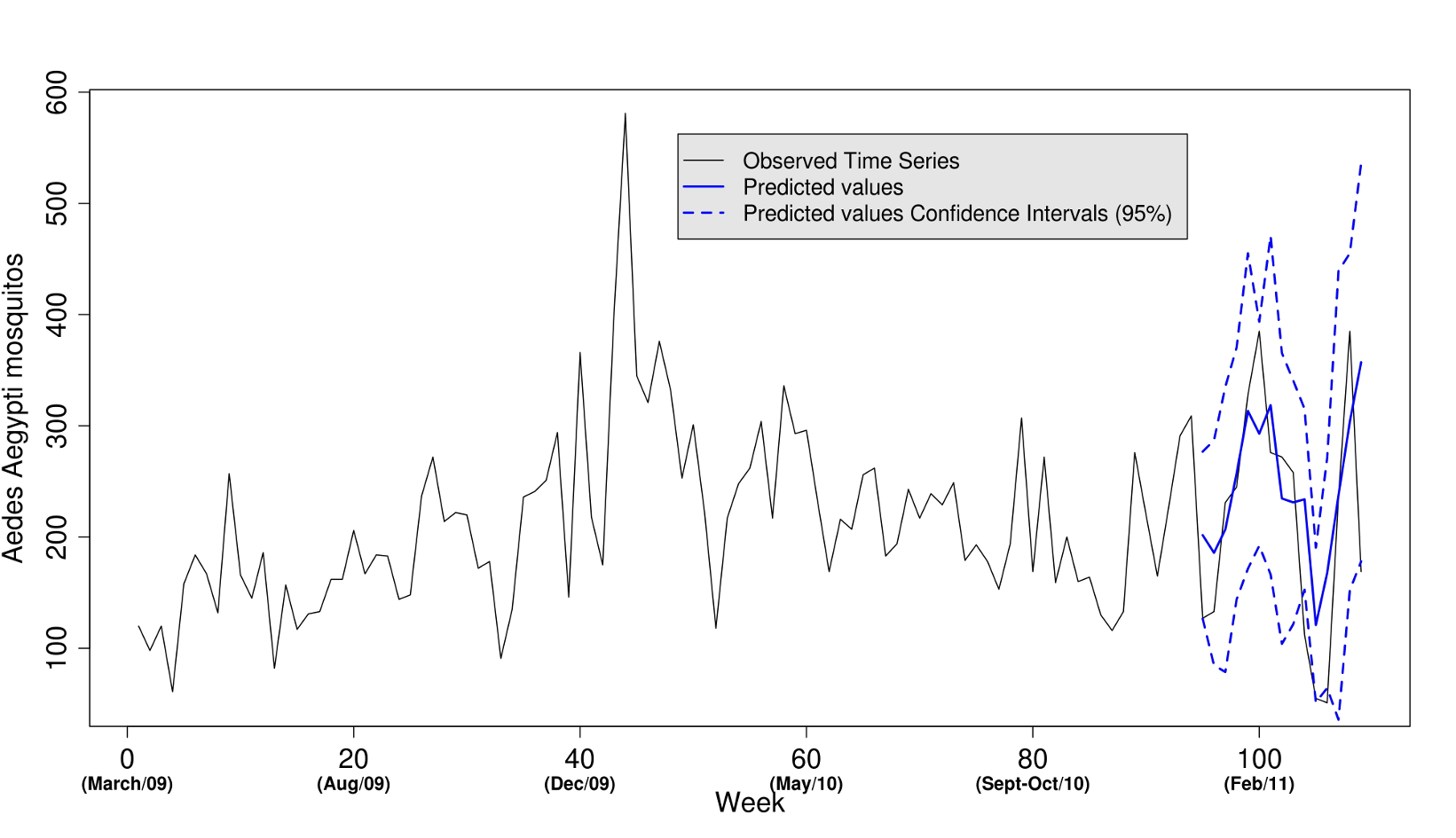

Supplement: Figure S4 — Predictability model. Graph of overlapping of values observed (series in black) of number of mosquitoes in the 109 weeks and forecast values (series in blue) to “out-of-fit” data. The dashed lines correspond to confidence intervals of 95% of forecast values. (TIFF) [file pone.0064773.s004.tiff]

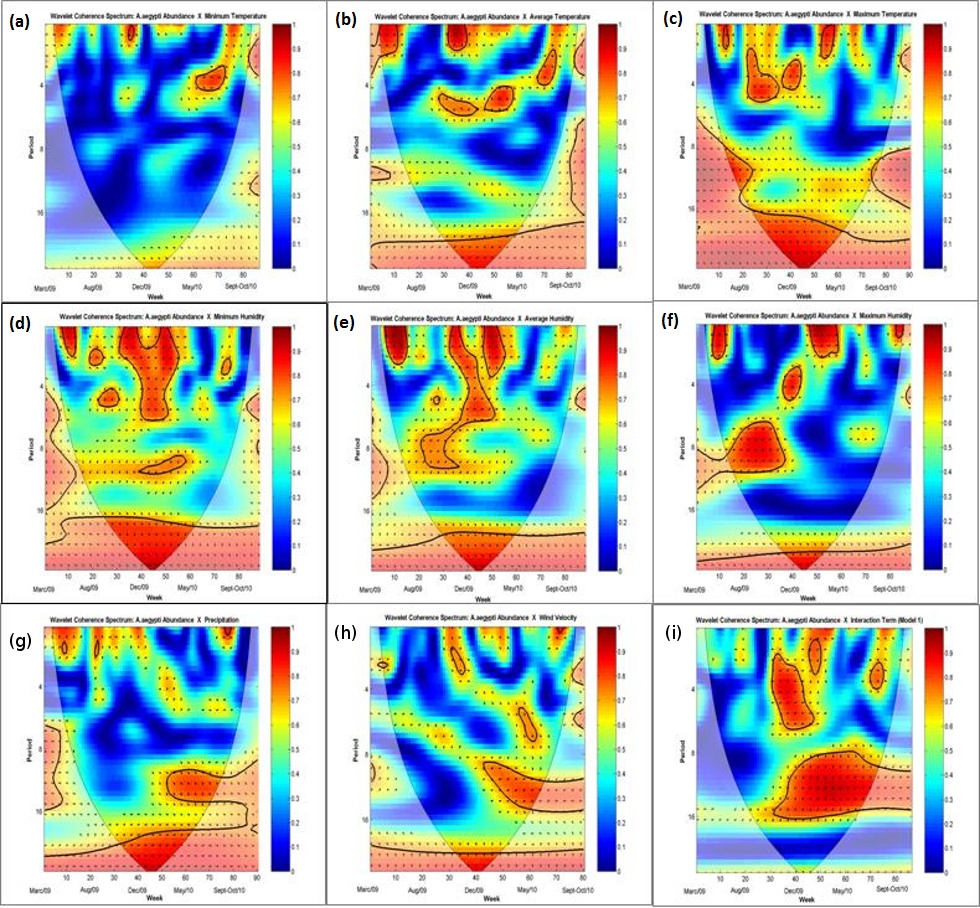

Supplement: Figure S5 — Wavelet Coherence Spectrums. Wavelet Coherence spectrum of Ae. aegypti Abundance/week/trap versus: First line: Temperature (Minimum, Average and Maximum). Second line: Humidity (Minimum, Average and Maximum). Third line: Precipitation; Wind Velocity; Term Interaction (Model 1). Blue, low coherence; red, high coherence. The black bold contours show α = 5% significance level. The cone of influence (black curve) indicates the region not influenced by edge effects. Period scale is in weeks. The y-axis is on a base 2 logarithmic scale. The black arrows represent the relative phase relationship (anti-clockwise direction starting at the west-east direction). In all graphs, the first series is the mosquito abundance and the second series is a meteorological variable: 0°: both series are in-phase; 45°: the second series is 1/8 of period ahead of the former, 90°: 1/4 of the period ahead; 135°: 3/8 of the period ahead; 180°: the series are out-phase; 225°: the second series is 3/8 of the period behind; 270°: 1/4 of the period behind, 315°: 1/8 of the period behind. (TIF) [file pone.0064773.s005.tif]

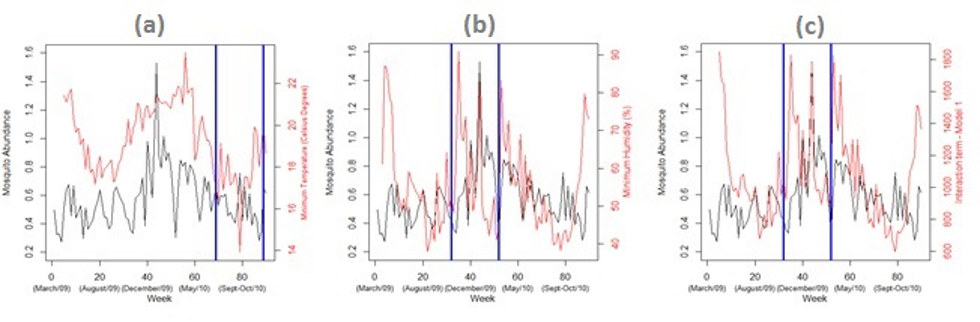

Supplement: Figure S6 — Meteorological and Mosquito Abundance series plot. Times series plots of the minimum temperature (left), minimum humidity (center) and de interaction term (right) time series matched with the mosquito abundance/week/trap. Blue vertical lines delimit the time period in which the association was significant in the wavelet analysis. (TIF) [file pone.0064773.s006.tif]
