# Supplementary material for: Modeling the Non-Stationary Climate Dependent Temporal Dynamics of Aedes aegypti
Source: PLoS One. 2013 Aug 20;8(8):e64773. doi: 10.1371/journal.pone.0064773 (PMC3748059; doi:10.1371/journal.pone.0064773)
Supplement: Table S1 — Model selection. Comparison of models with effects of the meteorological variables, autoregressive term and possible interactions of temperature and humidity (minimum, average and maximum) lagged weeks (lag) on mosquito abundance/week/trap. (DOCX) [file pone.0064773.s007.docx]

**Table S1 (Supporting Information).** **Model selection.** Comparison of models with effects of the meteorological variables, autoregressive term and possible interactions of temperature and humidity (minimum, average and maximum) lagged weeks (lag) on mosquito abundance/week/trap

| Model | AIC |
| --- | --- |
| **Model 1:** AR(1) + Minimum Temperature (4) * Minimum Humidity (2) | 922.27 |
| **Model 2:** AR(1) + Minimum Temperature (4) * Average Humidity (2) | 924.72 |
| **Model 3:** AR(1) + Minimum Temperature (4) * Maximum Humidity (2) | 929.64 |
| **Model 4:** AR(1) + Average Temperature (4) * Minimum Humidity (2) | 922.88 |
| **Model 5:** AR(1) + Average Temperature (4) * Average Humidity (2) | 924.87 |
| **Model 6:** AR(1) + Average Temperature (4) * Maximum Humidity (2) | 930.05 |
| **Model 7:** AR(1) + Maximum Temperature (0) * Minimum Humidity (2) | 937.62 |
| **Model 8:** AR(1) + Maximum Temperature (0) * Average Humidity (2) | 939.37 |
| **Model 9:** AR(1) + Maximum Temperature (0) * Maximum Humidity (2) | 946.02 |
