# Supplementary material for: Modeling the Non-Stationary Climate Dependent Temporal Dynamics of Aedes aegypti
Source: PLoS One. 2013 Aug 20;8(8):e64773. doi: 10.1371/journal.pone.0064773 (PMC3748059; doi:10.1371/journal.pone.0064773)
Supplement: Table S2 — Thresholds shift effects on mosquito abundance. Temperature and humidity thresholds shift effects on mosquito abundance/week/trap to the nine fitted models. (DOCX) [file pone.0064773.s008.docx]

**Table S2 (Supporting Information). Thresholds shift effect on mosquito abundance.** Temperature and humidity thresholds shift effects on mosquito abundance/week/trap to the nine fitted models

| **Interaction Term (row X column)** | **Minimum Humidity** | **Average Humidity** | **Maximum Humidity** |
| --- | --- | --- | --- |
| **Minimum Temperature** | 43.6%  15.7ºC | 65.0%  11.8ºC | 78.3%  8.4ºC |
| **Average Temperature** | 42.8%  18.1ºC | 61.6%  14.0ºC | 76.9%  9.7ºC |
| **Maximum Temperature** | 26.7%  23.3ºC | 42.6%  19.6ºC | 63.0%  14.6ºC |
