# Supplementary material for: Modeling the Non-Stationary Climate Dependent Temporal Dynamics of Aedes aegypti
Source: PLoS One. 2013 Aug 20;8(8):e64773. doi: 10.1371/journal.pone.0064773 (PMC3748059; doi:10.1371/journal.pone.0064773)
Supplement: Table S3 — Process of fitting the best model. Estimates of the gradual fit of the best model. (DOCX) [file pone.0064773.s009.docx]

**Table S3 (Supporting Information). Process of fitting the best model.** Estimates of the gradual fit of the best model.

| **Model: AR(1) – AIC = 1053.46** | | | |
| --- | --- | --- | --- |
| **Variable** | **Estimate** | **Standard Error** | **p-value** |
| Mosquito abundance (*lag*=1) | -0.6593 | 0.0750 | <2e-16 |
| **Model: Minimum Temperature + Minimum Humidity + Interaction Term – AIC = 947.68** | | | |
| **Variable** | **Estimate** | **Standard Error** | **p-value** |
| Minimum Temperature (*lag*=4) | -0.0300 | 0.0083 | 0.0003 |
| Minimum Humidity (*lag*=2) | -0.0362 | 0.0075 | 1.2e-06 |
| Interaction Term | 0.0019 | 0.0003 | 7.9e-09 |
